# Impact of time-of-flight PET on quantification accuracy and lesion detection in simultaneous ^18^F-choline PET/MRI for prostate cancer

**DOI:** 10.1186/s13550-018-0390-8

**Published:** 2018-05-31

**Authors:** Urs J. Muehlematter, Hannes W. Nagel, Anton Becker, Julian Mueller, Kerstin N. Vokinger, Felipe de Galiza Barbosa, Edwin E. G. T. ter Voert, Patrick Veit-Haibach, Irene A. Burger

**Affiliations:** 1 Department of Diagnostic and Interventional Radiology, University Hospital Zurich, Zurich, Switzerland; 2 Department of Nuclear Medicine, University Hospital Zurich, Zurich, Switzerland; 3 University Hospital Zurich, Zurich, Switzerland; 4 Department of Diagnostic Imaging, Sirio Libanes Hospital, Sao Paulo, Brazil; 5 University of Zurich, Zurich, Switzerland; 6 Department Joint Medical Imaging, Toronto General Hospital, Toronto, ON Canada; 7 University of Toronto, Toronto, ON Canada

**Keywords:** PET/MRI, Attenuation correction, Time-of-flight, Prostate cancer

## Abstract

**Background:**

Accurate attenuation correction (AC) is an inherent problem of positron emission tomography magnetic resonance imaging (PET/MRI) systems. Simulation studies showed that time-of-flight (TOF) detectors can reduce PET quantification errors in MRI-based AC. However, its impact on lesion detection in a clinical setting with ^18^F-choline has not yet been evaluated. Therefore, we compared TOF and non-TOF ^18^F-choline PET for absolute and relative difference in standard uptake values (SUV) and investigated the detection rate of metastases in prostate cancer patients.

**Results:**

Non-TOF SUV was significantly lower compared to TOF in all osseous structures, except the skull, in primary lesions of the prostate, and in pelvic nodal and osseous metastasis. Concerning lymph node metastases, both experienced readers detected 16/19 (84%) on TOF PET, whereas on non-TOF PET readers 1 and 2 detected 11 (58%), and 14 (73%), respectively. With TOF PET readers 1 and 2 detected 14/15 (93%) and 11/15 (73%) bone metastases, respectively, whereas detection rate with non-TOF PET was 73% (11/15) for reader 1 and 53% (8/15) for reader 2. The interreader agreement was good for osseous metastasis detection on TOF (kappa 0.636, 95% confidence interval [CI] 0.453–0.810) and moderate on non-TOF (kappa = 0.600, CI 0.438–0.780).

**Conclusion:**

TOF reconstruction for ^18^F-choline PET/MRI shows higher SUV measurements compared to non-TOF reconstructions in physiological osseous structures as well as pelvic malignancies. Our results suggest that addition of TOF information has a positive impact on lesion detection rate for lymph node and bone metastasis in prostate cancer patients.

**Electronic supplementary material:**

The online version of this article (10.1186/s13550-018-0390-8) contains supplementary material, which is available to authorized users.

## Background

Direct combination of magnetic resonance imaging (MRI) with positron emission tomography (PET) is a recent advance in hybrid imaging, and the demand for such imaging is continuously growing. Early clinical experience showed comparable results of PET/MRI in the detection of malignant lesion compared to PET/computed tomography (CT) [1]. However, a major advantage of hybrid PET/MRI is the combination of high soft tissue contrast and multi-parameter images from MR with functional and molecular information from PET, which could be especially beneficial in the pelvis, as was suggested by several authors [2–4].

In high-risk patients or patients with the suspicion of extra pelvic diseases, use of PET/CT with either ^18^F-choline or more recently also ^68^Ga-PSMA is widely used and showed improved accuracy compared to morphologic imaging alone [5]. Studies comparing ^18^F-choline PET/CT and PET/MRI yielded highly comparable results concerning lesion detection and choline uptake in patients with prostate cancer [6–9] with the benefit of improved anatomical localization [7]. Furthermore, the advantages of improved tracer localization in Choline PET/MRI could even improve accuracy of local staging over multiparametric MRI alone [10–12]. Therefore, PET/MRI is a highly promising field for prostate cancer.

Attenuation correction (AC) of PET remains an inherent problem of quantitative PET/MRI imaging since it is based on either an atlas-based (brain) or a direct MR image segmentation-based AC neglecting metal or bone density. Newer template-based AC algorithms including pattern recognition/machine learning and transmission/emission-based methods have recently been incorporated into magnetic resonance-based attenuation correction (MRAC) but are not clinically available [13, 14]. It has been demonstrated that segmentation-based MRAC, which is the standard method on most commercial PET/MRI scanners, treating bone as soft tissue, leads to substantial underestimation of SUV in bone lesions [15–20]. A recent simulation study showed that TOF PET can reduce MRAC-induced quantification errors in bone tissue [21]. However, it remains unclear if this reduced MRAC-induced bias on SUV measurements has clinical consequences for ^18^F-choline PET/MRI in prostate cancer patients.

An important improvement in PET was the development of the time-of-flight (TOF) function which was initially introduced in the clinical setting in the 1980s [22]. Since then, PET technique has substantially improved resulting in fully-3D TOF PET scanners available today [23]. With the development of new PET detector systems, there are now fully integrated PET/MRI systems available featuring TOF. Previous studies have shown that TOF information improves image quality of PET/CT [24–28] and PET/MRI [29–31]. For example, the implementation of TOF in PET/MRI notably reduces image acquisition time [32], metal artifacts [31, 33–35], as well as artifacts caused by respiratory mismatch between emission and data [21, 36]. Using the same TOF PET images from PET/CT with MRI or CT in a trimodality setting showed that the PET/CT and PET/MRI performed comparable in whole-body oncology [37], but for PET/MRI, the addition of TOF improved lesion quantification [38]. However, studies comparing TOF and non-TOF PET/MRI addressing specific tumor entities are still rare.

Thus, the purpose of our study was to examine the impact of TOF versus non-TOF reconstruction for ^18^F-choline PET/MRI regarding absolute and relative SUV_max_ differences and metastasis detection in the setting of prostate cancer patients.

## Methods

### Subjects

Twenty men referred to our department for an ^18^F-choline PET/CT were prospectively enrolled in this study from December 2014 to January 2016.There was no further selection applied regarding patient inclusion. Exclusion criteria were (a) refusal to study participation or (b) claustrophobia or other contraindications for MRI (e.g., cardiac pacemakers). Informed consent was obtained from each patient prior to the study inclusion and to the PET/MRI. The local ethics committee approved the study under reference number KEK ZH-Nr 2013-0220.

### PET/MRI protocol

All patients underwent a single injection of ^18^F-choline (mean dose ± standard deviation, 201 ± 4.7 MBq, range 195–214 MBq). Whole-body PET/MRI was performed after 59.3 min ± 4.9 min (mean ± SD) after injection. These PET/MRI study scans were acquired with a simultaneous TOF PET/MRI system (SIGNA PET/MR, GE Healthcare, Waukesha, WI, USA) used in previous studies at our department [33]: The scanner comprises a 3T wide-bore MR system with a TOF-PET detector ring installed between the body and gradient coils. The trans-axial and axial fields of view are 600 and 250 mm, respectively. The resolving time of the TOF detector is less than 400 ps [39].

The default acquisition protocol consisted of six bed positions (2 min per bed position), from the vertex of the skull to the mid-thighs. During PET scanning, MRI acquisitions for attenuation correction were performed, using in-phase and out-of-phase images to calculate water-only and fat-only images according to Dixon’s method [40, 41]. Additionally, the basic whole-body protocol and dedicated sequences to the pelvic region were applied. The MRI protocols and parameters are listed in Table 1.

**Table 1:** MRI protocol parameters

	Axial LAVA-FLEX^a^	Coronal T2w FRFSE-XL^b^	Axial T2w FRFSE-XL (Pelvic)	Coronal T2w FRFSE-XL (Pelvic)	Axial DWI^c^ EPI^d^ (Focus) (Pelvic)
Repetition time, TR (ms)	5618	6326	2600	2900	4000
Echo time, TE (ms)	2.66	118	118	121	67.2
Flip angle, FA (degrees)	12	111	125	125	90
Acquisition matrix	344 × 256	288 × 224	416 × 224	416 × 224	160 × 80
Image size (voxels)	512 × 512	512 × 512	512 × 512	512 × 512	256 × 256
Reconstruction diameter (mm)	460	480	180	200	240
Slice thickness (mm)	3 (3D)	5	4	4	4
Signal averages	0.68	1	4	4	–
b-values (s/mm2) and signal averages	–	–	–	–	0 (6 av.)
					400 (8 av.)
					700 (16 av.)
Diffusion direction	–	–	–	–	‘All’
Bandwidth (Hz/pixel)	651	355	326	326	1953
Acquisition time (mm:ss)	0:18	0:52	3:39	4:04	5:16

^a^3D, fast spoiled gradient echo imaging technique that generates water only, fat only, in phase, and out of phase echoes in one single acquisition (GE Healthcare, Waukesha, WI, USA)

^b^Fast relaxation fast spin echo

^c^Diffusion-weighted imaging

^d^Echo-planar imaging

### MR imaging-based attenuation correction

An atlas-based attenuation correction was used for the head [42]. For the remaining body regions, air, lung, and soft tissue were segmented and a continuous fat-water based MRAC method was applied [43, 44].

### TOF and non-TOF PET reconstructions and image analysis

3D-PET emission data was reconstructed with TOF and non-TOF for each patient with a fully three-dimensional iterative algorithm, which is part of the manufacturer-supplied standard scanner software (ordered subset expectation maximization (OSEM)-based VUE Point FX for TOF PET and VUE Point HD for non-TOF PET, GE Healthcare, Waukesha, WI, USA). Both algorithms include standard scatter, dead-time, random attenuation, and normalization correction as well as correction for the detector response using Sharper (GE Healthcare, Waukesha, WI, USA). Their only difference lies in the TOF information, which is ignored by the VUE Point HD algorithm. The PET images for both TOF and non-TOF were reconstructed using 2 iterations, 28 subsets, a 600-mm field of view, and a 256 × 256 image grid with 2.34 × 2.34 ×  2.78 mm^3^ voxels. For image space filtering, an in-plane Gaussian convolution kernel with a full width at half maximum of 4.0 mm was used, followed by a standard axial filter with a 3-slice kernel.

Reconstructed images were reviewed on a dedicated workstation (Advantage Workstation, Version 4.6, GE Healthcare, Milwaukee, WI, USA) for region of interest (ROI) analysis. Maximum standardized uptake values (SUV_max_) was normalized to patient body weight. SUV_max_ was calculated for nine physiological structures using rectangle ROIs of 20 × 20 mm: skull, C7, Th12, L5, femoral head, pelvis, kidneys, ischioanal fossa, and M. gluteus. Average values were calculated for symmetrical structures. Lesions in the prostate, lymph node, and bone were assessed with size-adapted ROIs with knowledge of the reference standard. Additionally, size of lymph node metastases (maximal short axis) as well as minimum distance to osseous tissue (lymph node surface to cortical bone) was measured on T2-weighted MR images.

Four readers (readers 1 and 2, board-certified radiologist with 2 and 1 years of fellowship training in Nuclear Medicine; reader 3, 2 years of residency training in Nuclear Medicine; reader 4, 3 years of residency training in Radiology and 1 year of fellowship training in Nuclear Medicine) reviewed all reconstructed images on a dedicated review workstation. Readers investigated non-TOF PET and 1–3 months later TOF PET and were blinded to any other information than the patient bodyweight and injected dose (necessarily needed for the SUV calculations). Readers 1–4 evaluated the data for image quality, prostate lesions detection (or prostate bed if post-prostatectomy), lymph node metastases detection, and osseous and visceral metastases detection. Image quality was ranked from 4 (excellent) to 0 (non-diagnostic), and it was evaluated based on general quality, image sharpness, noise, and presence of artifacts (noise was evaluated in a scale from 0 (high noise) to 4 (low noise), artifacts from 0 (no artifacts) to 2 (severe artifacts), respectively). Readers 1 and 2 furthermore evaluated exact lymph node and osseous metastases site. An unblinded read out (board-certified Nuclear Medicine Physician and Radiologist), including all available histopathology and clinical/imaging follow-up data served as a reference standard. Details of the reference standard are shown in Table 2.

**Table 2:** Reference standard of lesions

	Histology	Clinical/imaging follow-up
Lymph node metastases	10	9^a^
Osseous metastases	0	15^b^

^a^Median follow-up duration 276 days (range 188–364)

^b^Median follow-up duration 189 days (range 74–410)

### Statistical analysis

Difference of SUV_max_ (SUV_max n-TOF_ − SUV_max TOF_) was compared using a two-sample paired *t* test and to achieve an alpha of 0.05, a conservative multiple comparisons correction of Bonferroni was applied (*n* = 12 different ROIs compared in each patient) [45]. Significant difference was defined at *p* < 0.05. Relative difference of SUV_max_ was defined as (SUV_max n-TOF_ − SUV_max TOF_)/SUV_max TOF_ and was compared using Bland Altman plots [46]. Results are presented as mean and relative standard deviation (RSD). Agreement among observers was evaluated using Fleiss’s kappa (κ) [47, 48] for all readers. Interpretation of κ was based on a classification provided by Landis and Koch: 0.0, poor; 0.0–0.20, slight; 0.21–0.40, fair; 0.41–0.60, moderate; 0.61–0.80, good; 0.81–1.00, almost-perfect reproducibility [49].

The Spearman’s rank correlation was used to investigate the effect of lymph node lesion size and the distance between lymph node lesions and bone on relative SUV_max_ difference. Lesion detection percentage was calculated for lymph node metastasis and distant metastasis. The Wilcoxon signed-rank test was used to compare visual image quality scores between TOF and non-TOF reconstructions. Neither sensitivity nor specificity measurements were calculated due to the heterogenous patient population and application of a heterogenous reference standard. All analyses were conducted in R 3.2.5 (The R Software Foundation, Vienna, Austria).

## Results

A total of 20 patients with a mean age of 72.5 years (range 60–89 years) were consecutively included in our study. Of these, 11 were referred for restaging and 9 for initial staging of prostate cancer. Table 3 summarizes the demographic and clinically relevant details of our study population. A total of 241 ROIs were analyzed. One hundred eighty ROIs were set in physiological tissue, 25 ROIs in prostate lesions, 19 ROIs in lymph node metastases, and 17 ROIs in osseous metastases to calculate absolute and relative differences. Our study population included 19 lymph node metastases in nine patients. Seven patients had bone metastases, two with disseminated disease. The remaining five patients showed 15 osseous metastases. In two patients, no cause for an elevated PSA could be identified. An overview of absolute and relative differences for physiological tissue and lesions are given in Tables 4 and 5.

**Table 3:** Summary of patient characteristics

	*N*	% of *N*
Patients	20	
Age at scan in years (median, range)	72.5 (60–89)	
Body height in m (median, range)	1.74 (1.60–1.87)	
Body weight in kg (median, range)	80 (57–114)	
BMI^a^ in kg/m^2^ (median, range)	27.5 (19.0–34.2)	
Reason for referral		
Restaging	11	55
Initial staging	9	45
Tumor location		
No tumor detected	2	10
Prostate	14	70
Lymph node metastases	9	45
Osseous metastases	7	35

^a^Body mass index

**Table 4:** Average SUV_max_ and SUV_max_ difference (difference significant if *p* < 0.05)

Site	TOF	TOF SD^a^	Non-TOF	Non-TOF SD^a^	n-TOF - TOF	*p* value	Highest value
Skull bone	− 0.71	0.19	0.87	0.24	0.16	< 0.001	non-TOF
C7	3.88	0.81	3.51	0.81	− 0.37	0.050	NS
TH12	5.69	1.4	2.73	1.17	− 2.96	< 0.001	TOF
L5	4.99	1.48	3.56	1.32	− 1.43	< 0.001	TOF
Femoral head	0.98	0.68	0.53	0.61	− 0.45	< 0.001	TOF
Pelvic bone	4	1.26	3.3	1.24	− 0.7	0.003	TOF
Kidney	17.98	3.31	17	3.38	− 0.98	< 0.001	TOF
Ischioanal fossa	1.46	0.36	1.35	0.4	− 0.11	1	NS
M. gluteus	2.12	0.63	2.06	0.61	− 0.06	1	NS
Prostate lesion	8.42	2.41	7.34	2.44	− 1.08	< 0.001	TOF
Lymph node lesion	8.66	3.55	7.03	3.54	− 1.63	< 0.001	TOF
Osseous lesion	9.46	6.57	8.23	6.33	− 1.24	0.008	TOF

Units: g/ml

^a^Standard deviation

**Table 5:** Average relative SUV_max_ differences

Site	(n-TOF -TOF)/TOF	RSD
Skull bone	23%	0.16
C7	− 9%	0.12
TH12	− 52%	0.15
L5	− 28%	0.17
Femoral head	− 48%	0.23
Pelvic bone	− 18%	0.17
Kidney	− 6%	0.04
Ischioanal fossa	− 6%	0.2
M. gluteus	− 1%	0.21
Prostate lesion	− 13%	0.11
Lymph node lesion	− 22%	0.13
Osseous lesion	− 17%	0.10

*RSD* relative standard deviation

**Fig. 1:**
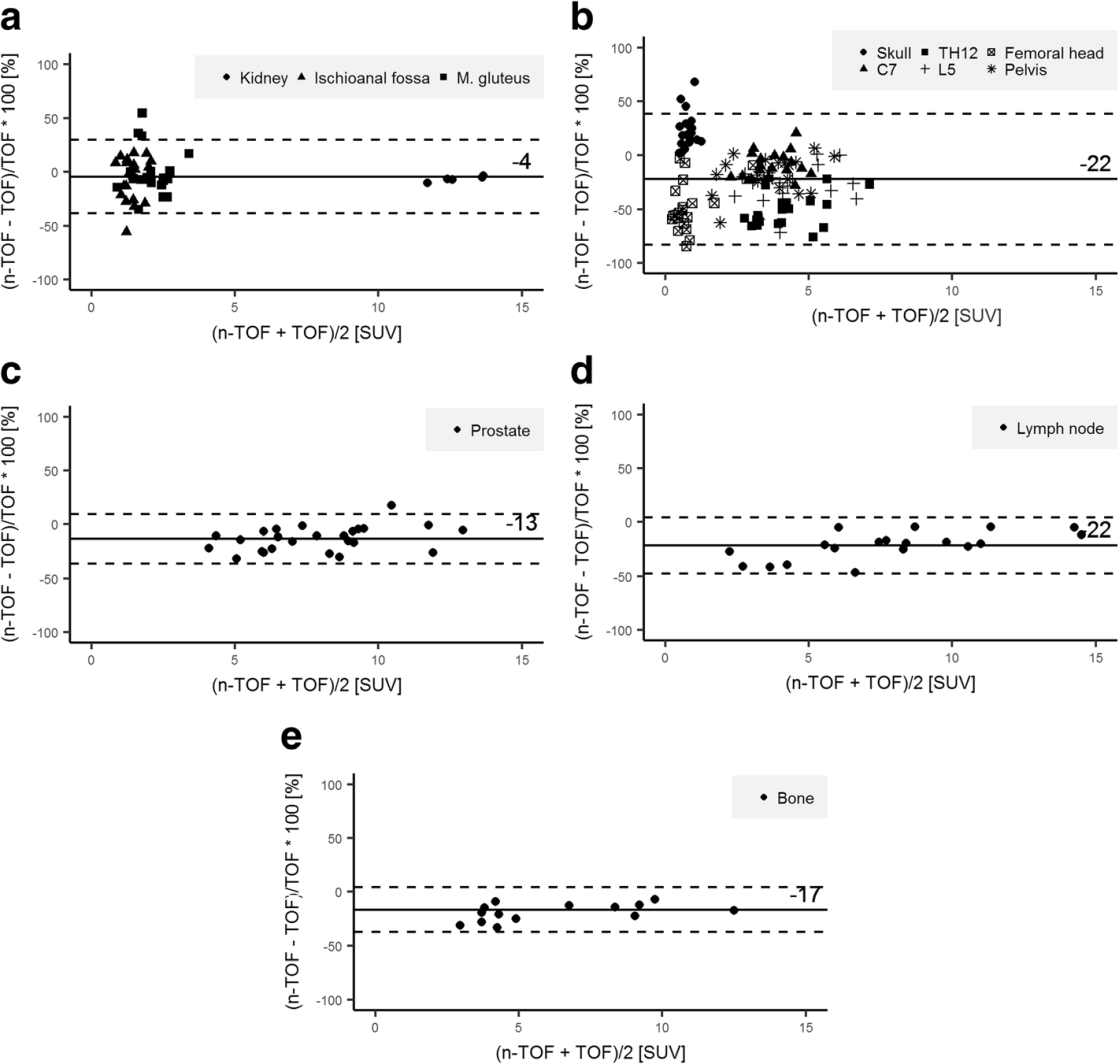
Bland-Altman plots with mean (bold line) and twice the standard deviation (dotted lines) of relative difference of maximum standard uptake value in soft tissue (**a**), bone (**b**), prostate cancer (**c**), lymph node metastases (**d**), and bone metastases (**e**). Note the good correlation of TOF and non-TOF reconstructions in soft tissue and the high relative difference in bone and lesions

**Fig. 2:**
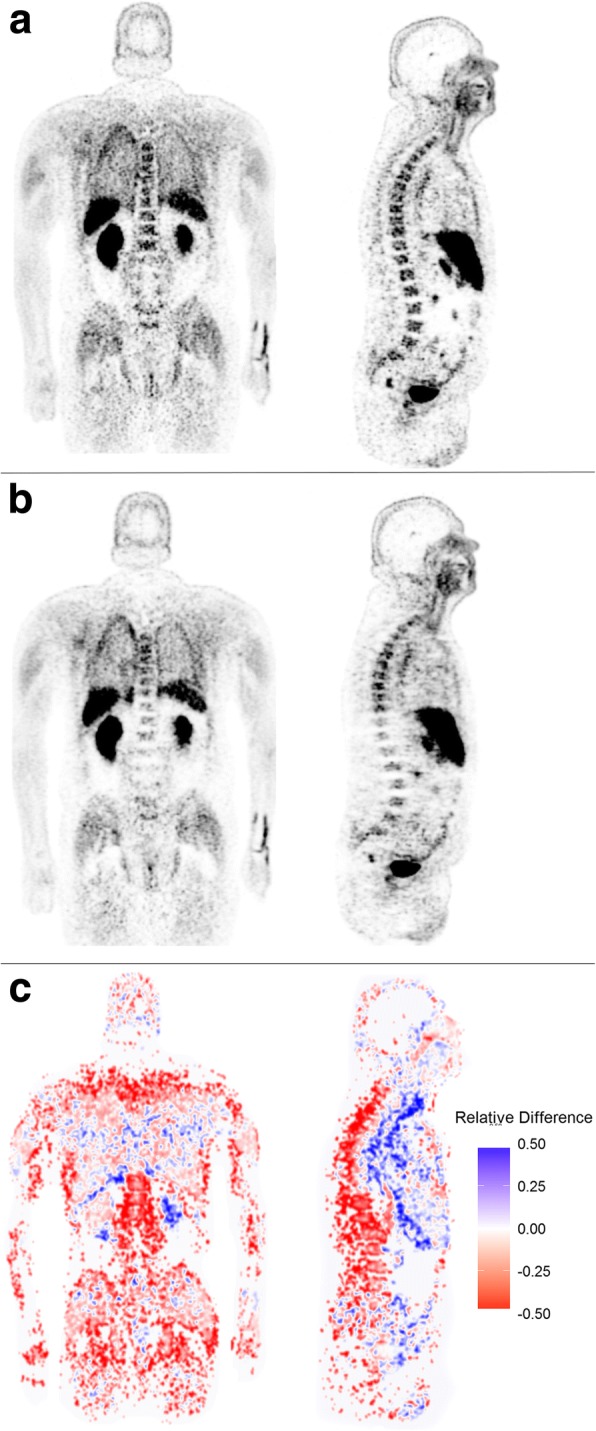
Coronal and sagittal TOF ^18^F-choline PET reconstruction (**a**). Coronal and sagittal non-TOF ^18^F-choline PET reconstruction (**b**). Relative percentage difference image (TOF - non-TOF)/TOF (**c**). Note the differences in the vertebra, the pelvic region, and the femora

SUV_max_ was significantly lower in non-TOF reconstructions compared to TOF images in all osseous structures, except in the bone of the skull with a mean relative difference of osseous structures without skull bone of − 31% (RSD ±23). However, there was no significant difference in the pooled physiological soft tissue data between non-TOF and TOF, although the difference was significant for the kidney alone. Typical distribution of relative differences in physiological tissue is shown in Bland-Altman plots in Fig. 1a, b. The highest relative difference of SUV_max_ was found in the Th12 and femoral heads with a mean relative difference of − 52% (RSD ± 15) and − 48% (RSD ± 23), respectively. Examples for relative difference images of TOF PET and non-TOF PET reconstructions for one patient are given in Fig. 2.

### Lesions

**Fig. 3:**
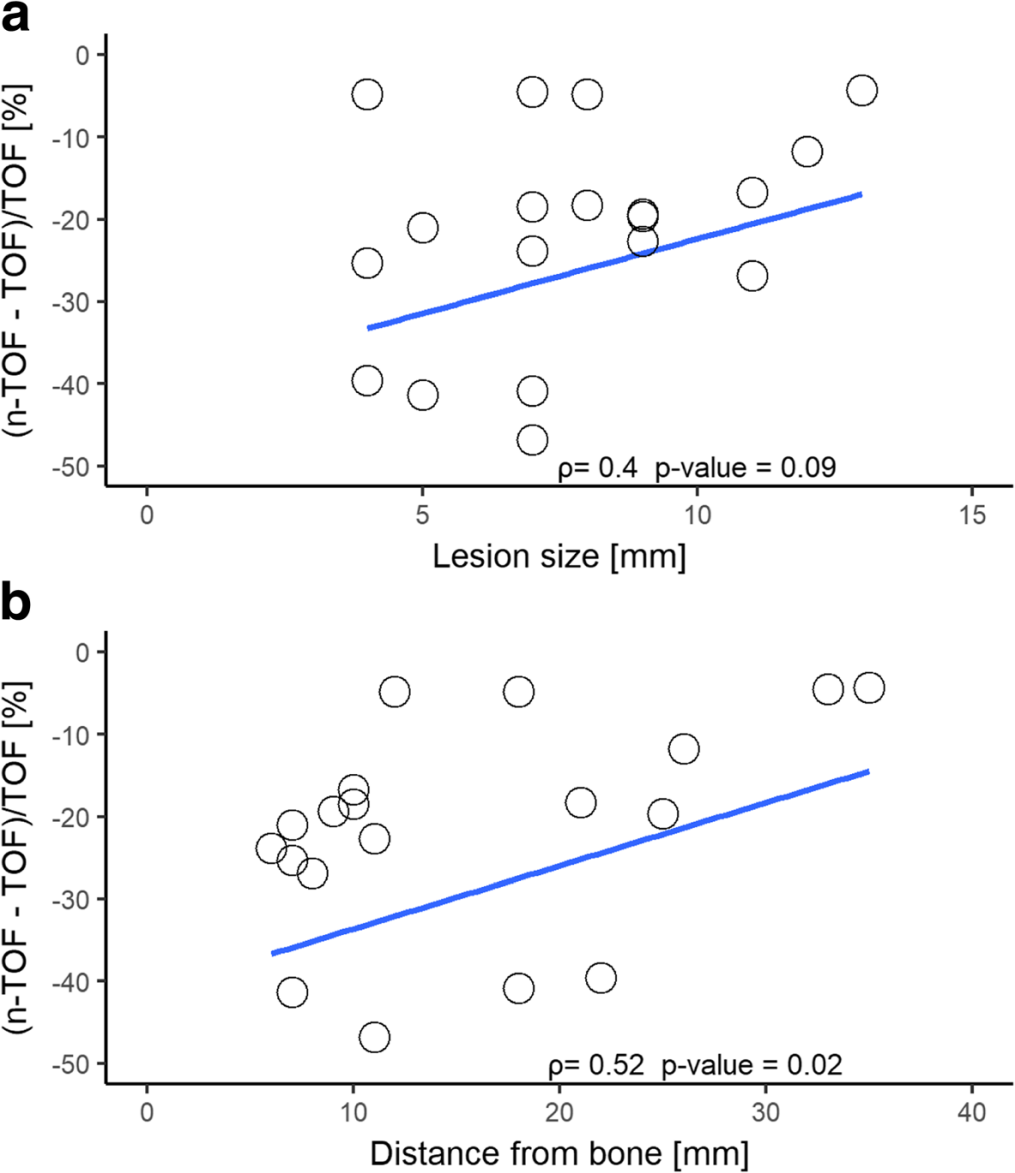
Relative maximum standard uptake value (SUV) differences for lymph node metastases according to lesion size (**a**) and distance of the lesion from the bone (**b**). Note the increasing relative difference with decreasing distance from the bone and decreasing lesion size. The line represents the Spearman’s rank correlation and *ρ* the Spearman’s rank correlation coefficient

SUV_max_ was significantly lower on non-TOF images for all lesions, with an overall underestimation of − 17% (− 13% for prostate lesions, − 22% for lymph node metastases, and − 17% for osseous metastases, respectively). Typical distribution of relative differences of lesions is shown in Bland-Altman plots in Fig. 1c, d, and e. Spearman’s rank correlation showed a negative correlation for both lymph node lesion size and their distance to bone to relative SUV_max_ difference (*ρ* = 0.4, *p* value = 0.09 and *ρ* = 0.52, *p* value = 0.02, respectively) as shown in Fig. 3.

**Fig. 4:**
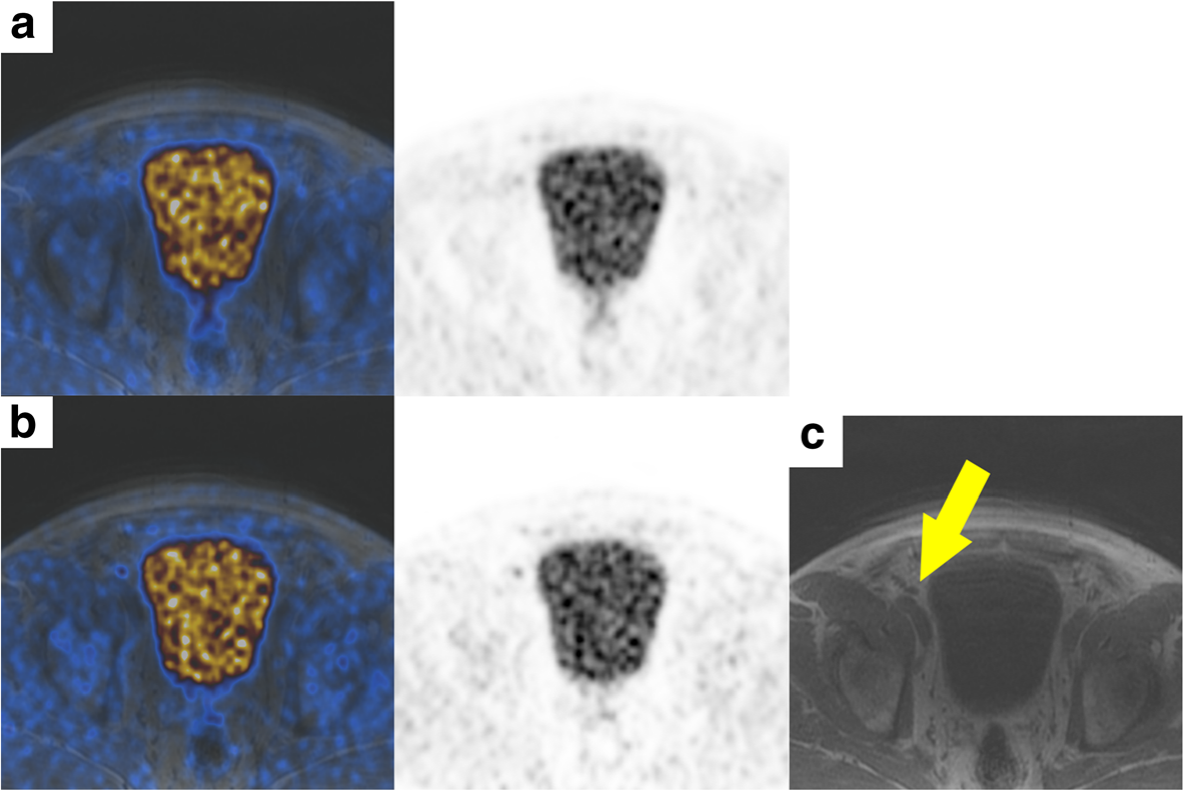
Images of a patient with a PET-positive lesion along the right external iliac vessel diagnosed as a lymph node metastasis of prostate cancer. **a** Fusion image (left) and PET image (right) of simultaneous ^18^F-choline non-TOF PET/MR. **b** Fusion image (left) and PET image (right) of simultaneous ^18^F-choline TOF PET/MR. **c** T1-weighted MR image showing a normal-sized lymph node (arrow)

Detailed results of all four readers are given in Table 6. Reader 1 detected 84% (16/19) of lymph node metastases on TOF PET reconstructions (three missed metastases with histopathology reference) and 58% (11/19) on non-TOF PET reconstruction (five missed metastases with histopathology, three with imaging/clinical follow-up as reference, respectively). Reader 1 detected 93% (14/15) of osseous metastases on TOF PET and 73% (11/15) on non-TOF PET reconstructions (all missed metastases with imaging/clinical follow-up as reference). Reader 2 detected 84% (16/19) of lymph node metastases on TOF PET reconstructions (two missed metastases with histopathology reference, one with imaging/clinical follow-up as reference, respectively) and 73% (14/19) on non-TOF PET reconstruction (three missed metastases with histopathology, two with imaging/clinical follow-up as reference, respectively). Reader 2 detected 73% (11/15) of osseous metastases on TOF PET and 53% (8/15) on non-TOF PET reconstructions (all missed metastases with imaging/clinical follow-up as reference). Example of a missed lymph node metastasis is given in Fig. 4. The interreader agreement between readers 1 and 2 was good for localization of lymph node metastases for TOF and non-TOF (TOF kappa = 0.652, 95% confidence interval [CI] 0.506–0.798 and non-TOF kappa = 0611, CI 0.465–0.757). The interreader agreement between readers 1 and 2 was almost perfect for localization of osseous metastases for TOF (TOF kappa = 0.81, CI 0.631–0.989) and good for non-TOF (non-TOF kappa = 0.756. CI 0.577–0.935). Interreader agreement between all reader was good for local cancer detection for both TOF and non-TOF (TOF kappa = 0.762, CI 0.583–0.941 and non-TOF kappa = 0.696, CI 0.517–0.875), respectively. For lymph node metastasis detection, the agreement was moderate for both TOF and non-TOF (TOF kappa 0.501, CI 0.323–0.680 and non-TOF kappa = 0.457, CI 0.278–0.636). For osseous metastasis detection, the interreader agreement was good for TOF (TOF kappa = 0.636, CI 0.453–0.810) and moderate for non-TOF (non-TOF kappa = 0.600, CI 0.438–0.780).

**Table 6:** Readout results

Nr.	Prostate cancer detection			Lymph node metastasis			Lymph node metastases count			Osseous metastasis			Osseous metastases count		
	Ref^a^	TOF (R1^b^/R2^c^/R3^d^/R4^e^)	Nn-TOF (R1^b^/R2^c^/R3^d^/R4^e^)	Ref	TOF (R1/R2/R3/R4)	Non-TOF (R1/R2/Rd./R4)	Ref	TOF (R1/R2/R3/R4)	Non-TOF (R1/R2/Rd./R4)	Ref	TOF (R1/R2/R3/R4)	Non-TOF (R1/R2/Rd./R4)	Ref	TOF (R1/R2/R3/R4)	Non-TOF (R1/R2/Rd./R4)
1	Y^f^	Y/Y/Y/Y	Y/Y/Y/Y	Y	N/N/N/Y	N/N/N/Y	1	0/0/0/1	0/0/0/1	N	N/N/N/Y	N/N/N/Y	0	0/0/0/1	0/0/0/1
2	N^g^	N/N/N/N	N/N/N/N	N	N/N/N/N	N/N/N/N	0	0/0/0/0	0/0/0/0	N	N/N/N/N	N/N/N/N	0	0/0/0/0	0/0/0/0
3	N	N/N/N/N	N/N/N/N	Y	Y/Y/Y/Y	N/Y/Y/Y	1	1/1/1/3	0/1/1/3	N	N/N/N/Y	N/N/N/N	0	0/0/0/6	0/0/0/0
4	Y	Y/Y/Y/Y	Y/Y/Y/Y	N	N/N/N/N	N/N/N/N	0	0/0/0/0	0/0/0/0	Y	Y/Y/Y/Y	Y/Y/Y/Y	6^h^	6/6/6/6	6/6/6/6
5	Y	Y/Y/N/Y	Y/N/N/Y	N	N/N/N/N	N/N/N/N	0	0/0/0/0	0/0/0/0	N	N/N/N/N	N/N/N/N	0	0/0/0/0	0/0/0/0
6	N	N/N/N/N	N/N/N/N	Y	Y/Y/Y/Y	Y/Y/N/N	2	2/3/2/2	1/2/0/0	Y	Y/Y/Y/Y	Y/Y/Y/Y	6	5/6/6/6	5/6/6/6
7	Y	Y/Y/Y/Y	Y/Y/N/Y	N	N/N/N/N	Y/N/N/N	0	0/0/0/0	1/0/0/0	N	N/N/N/N	N/N/N/N	0	0/0/0/0	0/0/0/0
8	Y	Y/Y/Y/Y	Y/Y/Y/Y	Y	Y/Y/Y/Y	Y/Y/N/Y	1	1/1/1/1	2/1/0/1	Y	Y/Y/N/Y	Y/N/N/Y	1	1/1/0/1	1/0/0/1
9	Y	Y/Y/Y/Y	Y/Y/Y/Y	N	N/Y/N/N	N/N/N/N	0	0/2/0/0	0/0/0/0	N	N/N/N/N	N/N/N/N	0	0/0/0/0	0/0/0/0
10	Y	Y/Y/Y/Y	Y/Y/Y/Y	Y	Y/N/N/N	Y/N/N/N	1	1/0/0/0	1/0/0/0	Y	Y/Y/Y/Y	Y/Y/Y/Y	6	6/6/6/6	6/0/6/6
11	Y	Y/Y/N/Y	Y/Y/Y/Y	N	Y/N/N/N	N/N/N/N	0	1/0/0/0	0/0/0/0	N	N/N/Y/N	N/N/Y/N	0	0/0/1/0	0/0/1/0
12	Y	Y/Y/Y/Y	Y/Y/N/Y	Y	Y/Y/Y/Y	Y/Y/Y/Y	6	6/6/6/6	5/6/6/6	Y	Y/Y/Y/Y	Y//Y/Y	5	4/3/1/3	4/6/1/3
13	Y	Y/Y/Y/Y	Y/Y/Y/Y	N	N/N/N/N	N/N/N/N	0	0/0/0/0	0/0/0/0	N	N/N/N/N	N/N/N/N	0	0/0/0/0	0/0/0/0
14	N	N/N/N/Y	N/N/N/Y	N	N/Y/N/Y	N/N/N/Y	0	0/2/0/2	0/0/0/2	Y	Y/N/N/N	N/N/Y/N	2	2/0/0/0	0/0/1/0
15	N	N/N/N/N	N/N/N/N	N	N/Y/N/N	N/N/N/N	0	0/1/0/0	0/0/0/0	N	N/N/N/N	N/N/N/N	0	0/0/0/0	0/0/0/0
16	N	N/Y/N/N	N/Y/N/N	Y	Y/Y/Y/Y	Y/Y/Y/Y	4	3/4/1/5	2/3/2/2	Y	Y/Y/N/N	Y/N/N/N	2	2/2/0/0	1/0/0/0
17	Y	Y/Y/Y/Y	Y/Y/Y/Y	N	Y/N/N/N	N/N/N/N	0	1/0/0/0	0/0/0/0	N	N/N/N/N	N/N/N/N	0	0/0/0/0	0/0/0/0
18	Y	Y/Y/Y/Y	Y/Y/Y/Y	N	N/N/N/N	N/N/N/N	0	0/0/0/0	0/0/0/0	N	N/N/N/N	N/N/N/N	0	0/0/0/0	0/0/0/0
19	Y	Y/Y/Y/Y	Y/Y/Y/Y	Y	Y/N/Y/Y	N/N/Y/Y	1	1/0/2/3	0/0/2/2	N	N/N/N/N	N/N/N/N	0	0/0/0/0	0/0/0/0
20	Y	Y/Y/Y/Y	Y/Y/Y/Y	Y	N/Y/N/Y	N/N/N/N	1	0/3/0/3	0/0/0/0	N	N/N/N/N	N/N/N/N	0	0/0/0/0	0/0/0/0

^a^Reference

^b^Reader 1

^c^Reader 2

^d^Reader 3

^e^Reader 4

^f^Yes

^g^No

^h^Multiple (> 5)

### Image quality

**Fig. 5:**
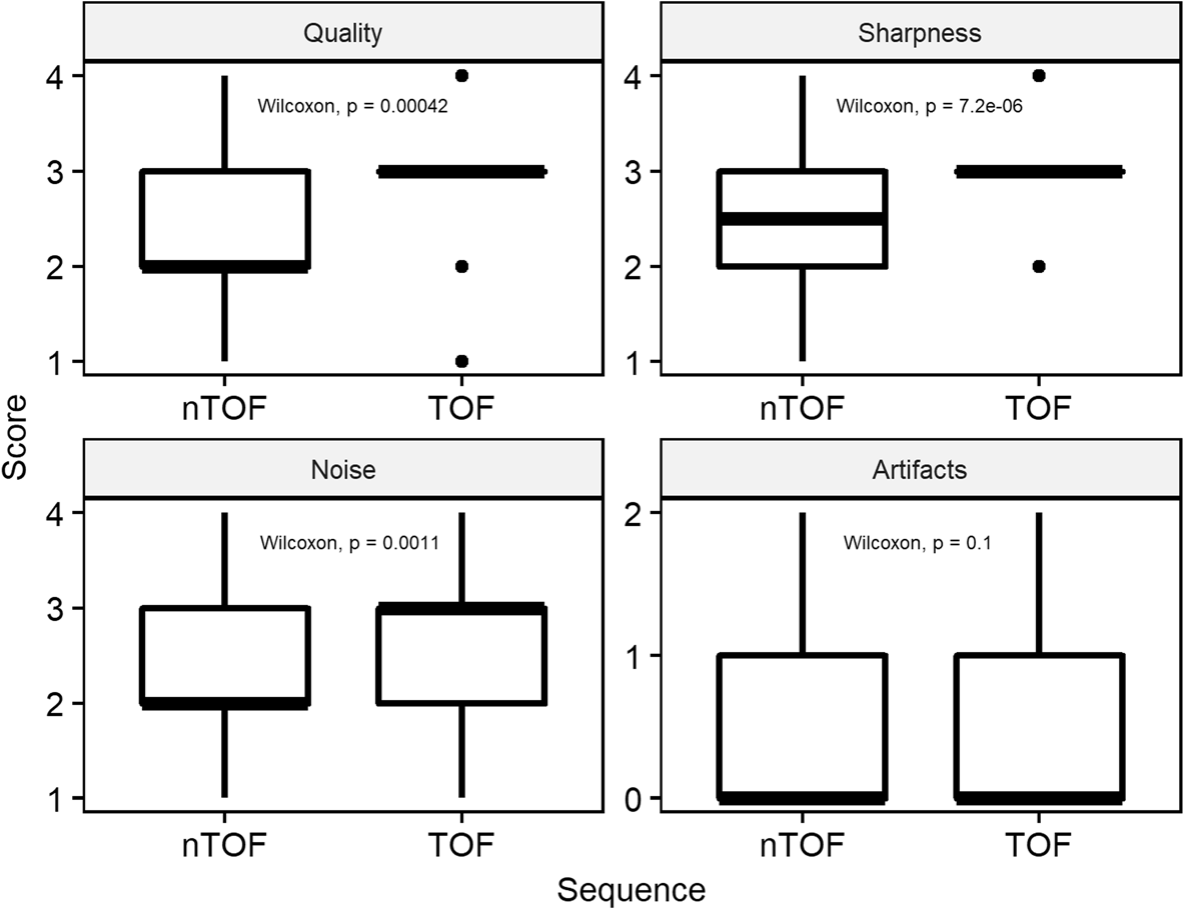
Image quality rating of TOF and non-TOF sequences

General image quality, image sharpness, and image noise were rated statistically significantly superior (*p* value < 0.001 for general quality and sharpness, *p* value < 0.05 for noise) on TOF-PET reconstructions compared to non-TOF-PET reconstructions, there was no significant difference in the rating of image artifacts (*p* value = 0.25) (Fig. 5). Mean score (and SD) for TOF and non-TOF sequences were 3.0 (± 0.7) and 2.6 (± 0.7) for general quality, 3.1 (± 0.6) and 2.6 (± 0.7) for image sharpness, 2.7 (± 0.7), 2.3 (± 0.7) for image noise, and 0.4 (± 0.6) and 0.5 (± 0.6) for artifacts. Details of all readers are given in Additional file 1: Table S1.

## Discussion

In the present study, we investigated the impact of non-TOF versus TOF reconstruction in ^18^F-choline PET/MRI on lesion detection in a clinical setting. We demonstrated that not only SUV measurements are significantly affected, but also the lesion detection rate is lower if PET data is reconstructed without TOF information. Furthermore, we compared SUV measurements of TOF and non-TOF reconstructions in physiological osseous or pelvic tissue and could confirm lower SUV_max_ values as previously found in simulated data, suggesting that TOF can recover some of the lost PET signal in areas under-corrected by the MRAC that does not consider bone tissue.

Comparing TOF and non-TOF PET OSEM reconstructions is not straightforward as TOF also accelerates the convergence rate of the iterative algorithm. Briefly, this means that TOF reconstructions converge faster (requiring fewer iterations) to the “true” (or more accurate) SUV. As each iteration adds more noise to the image, fewer iterations also means lower image noise. Finally, this can be interpreted as an improved SNR or contrast recovery [23, 25]. This “TOF effect” increases with improved TOF timing resolutions [50].

To avoid unacceptably noisy PET images, the OSEM iterative process is usually stopped early. With the injected dose and scan time applied in this study, both the TOF and non-TOF OSEM reconstructions needed to be stopped after two iterations. A consequence of the early termination of the iterative process is that the reconstructed PET images may have non-uniform recovery of activity as different image parts may converge at different rates. Using the same number of iterations in both TOF and non-TOF OSEM means, considering the faster convergence rate of TOF OSEM, that some TOF OSEM reconstructed image parts have more accurate (higher) SUV compared to the non-TOF OSEM reconstructed images. These TOF effects are already known from research on PET/CT. The application of TOF in PET/MR has however additional effects as was briefly mentioned in the introduction and indicated in this study. Most of the previous comparison regarding SUV measurements between TOF and non-TOF reconstructions has been done in PET/CT and showed improved PET quantification using TOF [51–53]. There is only little evidence that TOF reconstruction in PET/CT does not have a clinically relevant impact on SUV measurement [54]. Our data demonstrate that TOF PET/MRI reconstruction results in higher SUV_max_ measurements in physiological osseous structures as well as in lesions within the prostate, bones, and lymph nodes compared to non-TOF PET reconstructions. This is in concordance to preliminary data shown in an abstract by Mollard et al. who also found higher SUV_max_ in TOF reconstructions compared to non-TOF reconstructions in lesions in prostate patients with ^18^F-choline [55] and is consistent with the findings of a simulation study of Mehranian et al. [21].

Moreover, our results suggest that lymph node and osseous metastases detection rate is higher on TOF PET reconstructions compared to non-TOF PET reconstructions. Most of the previous patient studies evaluating lesion detection comparing TOF and non-TOF PET were achieved in PET/CT and suggested a better lesion detection for TOF reconstructions [25, 26, 56–59]. Hausmann et al. [58], for example, found a higher lesion detection rate in a study comparing TOF and non-TOF ^18^F-choline in PET/CT in 32 prostate cancer patients with biochemical recurrence. The gain in lesion detection due to TOF in PET/CT is attributed to several factors, whereas an improved signal to noise ratio and contrast to noise ratio is considered to play an important role [35, 60], especially in small lesions [57, 61, 62]. It is obvious that these factors are also relevant for PET/MRI, while the correction of SUV underestimation induced by MRAC plays an additional important role. Our measurements suggest that SUV underestimation does not only affect bone tissue but also lesion in the proximity of bones, leading to a negative correlation between relative difference in lymph node activity and distance to osseous structures, as shown in Fig. 3. An additional factor that could have influenced lesion detection is reduced scatter correction artifacts around the bladder and the liver/kidney in TOF, as previously described by Minamimoto et al. [35] in PET/MR. Our results additionally showed a higher interreader agreement for lymph node and bone metastasis on TOF compared to non-TOF.

A direct comparison of lesion detection in TOF and non-TOF PET/MRI in patients has not yet been reported. Therefore, no direct comparison of our results is possible. However, performance of choline PET/MRI compared to PET/CT in prostate patients has been investigated previously in several studies using both TOF capable PET/MRI [8, 9] and non-TOF PET/MRI [7, 12]. One study using TOF PET/MRI showed a higher lesion detection rate compared to TOF PET/CT [8], while two had equivocal results [9, 12] and one comparison of TOF PET/CT with non-TOF PET/MRI had a slightly higher detection rate on the TOF PET/CT (three lymph nodes, one bone lesion) [7].

TOF plays an important role in current PET/MRI imaging for standard MR imaging-based attenuation correction. Furthermore, TOF information is essential for promising emission-based attenuation correction techniques that might become standard attenuation correction in PET/MRI in the near future [50, 63–65]. All these improvements are important for an accurate PET quantification to improve lesion detection sensitivity and to establish comparable results among PET/CT and PET/MRI systems.

Although a TOF OSEM reconstruction takes approximately twice as long as a non-TOF OSEM reconstruction (3 m: 30 s vs 1 m: 45 s per bed on our system), the effect on the clinical workflow is usually limited. Modern reconstruction computers have sufficient computing power to reconstruct an average dataset in the same time as an average patient scan. As modern reconstruction computers and software are optimized for parallel processing on central processing unit (GPU) and/or GPU cores, they are scalable to most clinical requirements [60].

Our study has inherent limitations; the study population including only 20 and rather heterogeneous patients does not allow any conclusion on the overall accuracy of ^18^F-choline PET/MRI for staging or restaging prostate cancer, especially also given the heterogeneous reference standard with histopathology not available for all PET-positive lesions. However, bone metastases are usually not biopsied, and since it was our main interest to show the effect of TOF versus non-TOF on bony structures and metastasis, we selected a high-risk patient cohort for this project. Consequently, only a minority underwent surgical treatment or histological confirmation of the PET findings. Therefore, we did not report on sensitivity or specificity measurement of ^18^F-choline PET/MRI but rather pointed out the differences for qualitative and quantitative assessments of TOF versus non-TOF reconstructions. Our reported subjective image quality data might be biased due to the inherent problem that readers usually identify TOF and non-TOF reconstructions by its appearance in a blinded readout.

## Conclusion

Our results show that TOF reconstruction of ^18^F-choline PET/MRI increases SUV measurements in physiological osseous structures as well as pelvic malignancies compared to non-TOF reconstructions, especially in bony lesions or lymph nodes in the proximity of bones. Furthermore, our study suggests a positive impact on lesion detection rate for lymph node and bone metastasis in prostate cancer patients if TOF reconstruction is applied to ^18^F-choline PET/MR.

## Additional file

Additional file 1: **Table S1.** Results of image quality rating. (DOCX 12 kb)

## Abbreviations

AC: Attenuation correction
CT: Computed tomography
MR: Magnetic resonance
MRAC: Magnetic resonance-based attenuation correction
OSEM: Ordered subset expectation maximization
PET: Positron emission tomography
PET/MRI: Positron emission tomography magnetic resonance imaging
ROI: Region of interest
RSD: Relative standard deviation
SUV: Standard uptake value
SUVmax: Maximum standardized uptake value
TOF: Time-of-flight
