# Correction to: impact of time-of-flight PET on quantification accuracy and lesion detection in simultaneous ^18^F-choline PET/MRI for prostate cancer

**DOI:** 10.1186/s13550-018-0413-5

**Published:** 2018-07-27

**Authors:** Urs J. Muehlematter, Hannes W. Nagel, Anton Becker, Julian Mueller, Kerstin N. Vokinger, Felipe de Galiza Barbosa, Edwin E. G. T. ter Voert, Patrick Veit-Haibach, Irene A. Burger

**Affiliations:** 1 Department of Diagnostic and Interventional Radiology, University Hospital Zurich, Zurich, Switzerland; 2 Department of Nuclear Medicine, University Hospital Zurich, Zurich, Switzerland; 3 University Hospital Zurich, Zurich, Switzerland; 4 Department of Diagnostic Imaging, Sirio Libanes Hospital, Sao Paulo, Brazil; 5 University of Zurich, Zurich, Switzerland; 6 Department Joint Medical Imaging, Toronto General Hospital, Toronto, ON Canada; 7 University of Toronto, Toronto, ON Canada

## Correction

Unfortunately, after publication of this article [1], it was noticed that the name of Urs J. Muehlematter was incorrectly displayed as Urs J. Mühlematter. The corrected author list can be seen above and the original article has been corrected to reflect this.
