# Supplementary material for: Impact of time-of-flight PET on quantification accuracy and lesion detection in simultaneous 18F-choline PET/MRI for prostate cancer
Source: EJNMMI Res. 2018 May 31;8:41. doi: 10.1186/s13550-018-0390-8 (PMC5981153; doi:10.1186/s13550-018-0390-8)
Supplement: Supplementary file 1 — Table S1. Results of image quality rating. (DOCX 12 kb) [file 13550_2018_390_MOESM1_ESM.docx]

**Additional file**

Table S1: Results of image quality rating

|  |  |  | | Reader | | |
| --- | --- | --- | --- | --- | --- | --- |
|  | Sequence | R1^a^ mean (± SD*) | R2^b^ mean (± SD) | | R3^c^ mean (± SD) | R4^d^ mean (± SD) |
| General Quality | non-TOF | 2.5 (±0.69) | 2.55 (±0.69) | | 3.1 (±0.64) | 2.2 (±0.59) |
|  | TOF | 3.0 (±0.73) | 3 (±0.79) | | 3.2 (±0.52) | 2.7 (±0.59) |
| Sharpness | non-TOF | 2.7 (±0.57) | 2.6 (±0.6) | | 3.1 (±0.76) | 2.0 (±0.32) |
|  | TOF | 3.1 (±0.72) | 3.2 (±0.62) | | 3.1 (±0.69) | 2.9 (±0.31) |
| Noise | non-TOF | 2.2 (±0.62) | 2.1 (±0.55) | | 2.9 (±0.67) | 2.1 (±0.55) |
|  | TOF | 2.5 (±0.76) | 2.75 (±0.79) | | 3.0 (±0.69) | 2.5 (±0.61) |
| Artifacts | non-TOF | 0.9 (±0.64) | 0.9 (±0.64) | | 0.15 (±0.37) | 0.1 (±0.31) |
|  | TOF | 0.6 (±0.75) | 0.65 (±0.75) | | 0.15 (±0.37) | 0.1 (±0.31) |

^a^, Reader 1; ^b^, Reader 2; ^c^, Reader 3; ^d^, Reader 4; *, Standard deviation
